# Animal transgenesis on the fast track in the era of evolving genome editing technologies: report for TT2017 the 14th Transgenic Technology Meeting in Snowbird, Utah

**DOI:** 10.1007/s11248-018-0060-7

**Published:** 2018-02-03

**Authors:** Yasuhide Furuta, Hiroshi Kiyonari

**Affiliations:** 1 RIKEN Center for Life Science Technologies, Kobe, Japan; 2 Memorial Sloan Kettering Cancer Center, New York, NY USA

TT2017, the 14th Meeting of the International Society for Transgenic Technologies (ISTT), was held at a secluded and majestic site of the Snowbird Ski Resort in Salt Lake City, Utah, USA, from October 1–4. The Meeting was hosted by the University of Utah Health with Susan Tamowski, Director of their Transgenic and Gene Targeting Core, as Chair of the Organizing Committee. Delegates visiting the Resort for the first time in early October preceding the approaching ski season may have felt the weather harshly cold for this time of the year with some snowfalls during the earlier half of the Meeting. Our colleagues from Japan went to a cafe for breakfast in a separate building before a snowfall, and at the end of their meal found themselves in the midst of a landscape completely transformed to white. It was a WARM welcome to remind us of rapid weather changes that can occur in the mountains of Utah. We also felt the effect of high altitude in a most personal way while walking up stairs. Later in the evening, immediately following the Opening Keynote Lecture, meeting delegates took the evening tram ride up to The Summit at Hidden Peak, a guest facility of the Resort at 11,000 ft (> 3350 m), where a Welcome Reception was held. All were thrilled by spectacular views of the surrounding Wasatch-Cache National Forest with a beautiful sunset as a backdrop in the west and a magnificent far stretching Rocky Mountains towards the east, further augmenting the celebratory atmosphere of the commencement of this exciting meeting. Dr. Mario Capecchi, the recipient of this year’s ISTT Prize, was among the crowd at this event. He was so kind to pose with many participants for memorable photos with this eminent scientist.

The Meeting commenced in the afternoon of Sunday, October 1, with a Welcoming Address by Susan Tamowski and ISTT President Jan Parker Thornburg of University of Texas, MD Anderson Cancer Center (Houston, TX, USA). Tamowski briefly introduced a history of Snowbird within Little Cottonwood Canyon that was initially explored and developed as a silver mining site almost a century before its development into a world-renowned ski resort. It was announced that there were over 400 attendees with about 40 talks and more than 100 poster presentations, bringing about enthusiastic anticipation and excitement for active discussions and interactions about to take place. As genome editing approaches have become commonplace and a standard methodology in transgenesis, an overwhelming number of talks and posters touched upon various aspects of the CRISPR technology. Perhaps a notable highlight of the Meeting in light of this still evolving technology was that a large number of presentations reported its successful applications to a greater spectrum of organisms, ranging from mosquitos to farm animals and non-human primates.

The scientific program began with a talk by Alex Joyner of Memorial Sloan Kettering Cancer Center (New York, USA). Her Opening Keynote Lecture gave an excellent overview of the recent innovations in transgenic technologies in the mouse from a developmental geneticist’s view. She discussed elegant applications of some of those technologies, such as inducible gene activation/inactivation and genetic lineage tracing, to explore the regenerative capability of the early postnatal mouse cerebellum.

The morning session of Day 2 started with another Keynote Lecture by Jin-Soo Kim of the Institute for Basic Science/Seoul National University (Seoul, Republic of Korea) who is well known for his eminent contributions to the development and application of genome editing tools using programmable nucleases. He introduced an impressive array of innovations in genome editing applicable to a wide variety of organisms, from animals to plants, making us excited about the potential of such technologies in benefiting human life in the not too distant future. Following Kim’s lecture, Session 1 revisited the aspects of traditional gene targeting. Wojtek Auerbach of Regeneron Pharmaceuticals (Tarrytown, NY, USA) challenged the audience with the question of whether ES cells are obsolete in the era of CRISPR. His “NO” answer was supported by a narrative of his approach using gene targeting in ES cells to make complex genetic alleles involving multiple loci and manipulations over large genomic regions that are still infeasible with current CRISPR injection into zygotes. He also reviewed the results of a survey on the use of zygote electroporation, indicating rapid increase in the use of this technology amongst the ISTT community. He ended with the question of whether zygote injection could become obsolete. Simon Titen of University of Utah (Salt Lake City, UT, USA) gave an excellent summary of a series of innovations over the years in classical gene targeting, including *loxP* sequence variants to provide flexibility in using the Cre/loxP system, recombinase-mediated cassette exchange (RMCE) to modify existing alleles, and reversible gene targeting alleles. Session 2 introduced novel CRISPR genome engineering tools and technologies. Channabasavaiah Gurumurthy, aka. Guru, of University of Nebraska (Omaha, NE, USA) introduced recent developments in CRISPR-mediated genome editing approaches as a session overview, followed by his own experience with an improved gene targeting approach in zygotes, referred to as Easi-CRISPR, which facilitates targeted introduction of kb-size elements by long single strand DNA (lssDNA) donors. Tomoji Mashimo of Osaka University (Suita, Osaka, Japan) spoke about his targeted insertion approach in zygotes also using lssDNA donors. His method, referred to as CLICK, proved to be highly effective in generating large-size inserts, which otherwise is still difficult to achieve by zygote electroporation. They have successfully applied this technique to generate a variety of conditional mutant alleles in rats. Hiroshi Arakawa of FIRC Institute of Molecular Oncology Foundation (Milan, Italy) was interested in mechanisms underlying longevity in various different organisms, and his long-term goals are to apply forward genetic screens in species currently not amenable to genetic approaches. After painstaking efforts, he devised an innovative method to generate comprehensive gRNA libraries from cDNA libraries, which would be particularly beneficial for the application of CRISPR technologies to those species with limited genomic information.

After lunch break, Poster Session 1 offered intermingling, information sharing and discussions among meeting participants. Session 3 was A Panel Discussion on “How to Run a Transgenic Core”, a traditional event of TT Meetings. Elizabeth Williams of the University of Queensland Biological Resources (Brisbane, Australia) who chaired this session had solicited and compiled questions from the ISTT members, and four panelists, Ernst-Martin Füchtbauer of Aarhus University (Aarhus, Denmark), Willie Mark of Memorial Sloan Kettering Cancer Center (New York, USA), Branko Zevnik of University of Cologne (Köln, Germany) and Kathy Krentz of University of Wisconsin (Madison, WI, USA) addressed these questions. Each panelist gave a brief introduction to his/her facility activities and featured efforts, and then discussion continued onto a variety of practical issues in core facility management. Difficult realities, concerns and compromises were shared by the participants, including funding for research and development activities, general problems and solutions in the usage of CRISPR technologies, defining the premises of responsibility of a core facility in delivering genome-edited animals to users, and some concerns on licensing issues of the CRISPR technology.

Session 4 spotlighted the recent progress in non-mammalian transgenesis, starting with a talk by Kate O’Conner-Giles of University of Wisconsin-Madison (WI, USA) about the use of CRISPR technologies in Drosophila to elucidate novel genes involved in neural circuit development and plasticity. Lissa Herron of Roslin Institute, University of Edinburgh (Midlothian, Scotland, UK) described her approach in using transgenic chickens to serve as a bioreactor for mass-production of valuable proteins in eggs. Another classic model organism not to be omitted is *C. elegans*. Matt Schwartz from the University of Utah (Salt Lake City, UT, USA) utilized a novel reagent tool kit referred to as SapTrap to streamline vector construction for targeted tag insertion and corresponding gRNA expression to scale up the throughput of isolating CRISPR genome-modified worms expressing tagged proteins. These talks underscore how CRISPR technology is accelerating innovations not only in mice but also in a variety of other widely used model organisms.

Towards the evening, TT2017 delegates were honored to welcome Dr. Mario Capecchi of the University of Utah (Salt Lake City, UT, USA) to the podium as the 11th ISTT Prize awardee. The Prize was presented by Jan Parker-Thornburg, Susan Tamowski, and COO of genOway, Yacine Cherifi, the sponsor for this prize. The award presentation was accompanied by an introduction of the achievements of Dr. Capecchi in establishing gene targeting in the mouse which led to the Nobel Prize in Medicine and Physiology in 2007. In addition, introductory remarks by Tamowski on her long-term interactions with Dr. Capecchi added an entertaining flavor with a personal touch. He focused his lecture on his ongoing research of Hox gene function. While Hox genes are well known as regulators of animal body patterning, the story had an unexpected twist with the implication that Hoxb8 plays a role in animal behavior. In the absence of apparent developmental phenotypes of *Hoxb8* mutant mice, the team embarked on behavior analysis, resulting in the realization of peculiar features of mutant mice that resembles trichotillomania in humans, a characteristic OCD-like symptom of hair pulling that often escalates to hair loss. Use of extensive conditional gene KO approaches, genetic cell lineage manipulations, and cell transplantation experiments eventually pinpointed the exact cell type requiring this unexpected genetic function of Hoxb8, namely the microglia serving to maintain neural function and behavior. This impressive series of studies portrayed a great example of how mouse genetics could contribute to the elucidation of possible cellular and molecular mechanisms underlying poorly understood human disease conditions. With great excitement from Dr. Capecchi’s Lecture in mind, delegates adjourned for evening Poster Session 2 for further scientific interactions.

The second full day of the Meeting started with Session 5 “Large Animal Transgenesis; Non-Human Primates”. Irena Polejaeva of Utah State University (Logan, UT, USA) updated the recent progress in production of sheep and goat models of human diseases, such as atrial fibrosis and cystic fibrosis, using a variety of sophisticated techniques that include somatic cell nuclear transfer and the CRISPR system. She also spoke about their attempt to create trans-chromosomal goat models; e.g., one carrying a human artificial chromosome transgene on a background deficient for the endogenous counterpart gene of interest (such as IgM) towards development of a humanized sheep as a novel agricultural resource. Ana Paula Mulet of the Institut Pasteur Montevideo (Montevideo, Uruguay) followed with an overview of ongoing sheep transgenesis approaches using a series of techniques, such as classical pronuclear injection, SCNT, RNAi and Lentivirus. The highlight of her talk was on more recent, promising progress using CRISPR technology to generate genetically engineered sheep strains for disease modeling and agricultural applications. One exemplary story was the development of the *HYAL2* mutant strain that may confer disease-resistance to a breed otherwise known to be susceptible to certain viral infectious diseases. She also emphasized the importance of cryopreservation techniques for the success of genetic livestock resource development. Another series of challenging efforts aimed at improving environmental adaptation of farm animals was raised in the talk by Tad Sonstegard of Acceligen/Recombinetics (Centreville, MD, USA). His presentation included their systematically designed efforts to achieve tropical adaptation of livestock to improve heat tolerance, combined with genetic manipulations to improve milk production and resistance to infectious diseases. Sen Wu of China Agricultural University (Beijing, China) gave an overview of the current status of the newly initiated large-scale national pig mutagenesis project in China. Their impressive and ambitious project plan is aimed at production of pigs with some 25,000 genes modified and their basic phenotypic characterization incorporated into a large open resource in the next 10 years. The last, but not the least, of the large transgenic animal model discussed was the case of genome-edited monkey for the study of Parkinson’s disease (PD) by Maria Emborg of the University of Wisconsin (Madison, WI, USA). In non-human primates, genetically engineered PD models are yet to be developed, hence her current studies are focused on LRRK2, encoded by the *PARK8* gene, often mutated in PD family cohorts. Her group is attempting to modify this gene in monkeys using CRISPR in iPS cells as well as in vivo. She also emphasized the importance of the established methods to validate the PD models in non-human primates.

The Ethics and the 3Rs session completed the morning. The first presentation by Jan Parker-Thornburg on behalf of Nicole Duffee of American Association for Laboratory Animal Science (AALAS) (Memphis, TN, USA) and the next by Nicola Osborne of Responsible Research in Practice (Horsham, West Sussex, UK) both articulated an alarming trend of the increasingly unfavorable public sentiment on the use of animals in research in both US and UK, respectively. Commonly emphasized in these discussions were the importance of proactive and passionate, but not emotional, outreach approaches from researchers to the lay public in order to increase transparency and awareness of the research and its significance. Boris Jerchow of University Medical Center Hamburg-Eppendorf (Hamburg, Germany) followed with a discussion of the dilemma that researchers face in balancing scientific triple constraints (impact, time, budget) with animal welfare regulations. In particular, under an increasing trend of reproducibility crisis in science, he urged researchers to develop an appropriate culture of care to achieve 4Rs (conventional 3Rs + reproducibility/reporting) by systematically implementing the flow from sound experimental/statistical plans, execution, and documentation, to reporting. The fourth talk was the award lecture for this year’s 3Rs Prize by Roger Askew of Ozgene (Lincon, MA, USA) for the development of the “goGermline” system to maximize ES cell germline transmission. This elegant design utilizes host embryos for ES cell injection that inherently carry a genetic background for sterility, drastically bumping up the likelihood of germline transmission of the ES-derived genome even from low ES-contributed chimeras. This system could significantly reduce the number of mice required for extensive crosses in cases of low germline transmission from a given ES cell line or clone, an asset that deserves the appreciation for its contribution to the realization of the 3Rs. An additional talk selected from abstracts in this session was by Lin Wu of Harvard University (Cambridge, MA, USA). She shared their experience of Genome Modification Facility on over 200 genome editing projects in mice using CRISPR techniques to successfully accommodate a wide range of research needs from small deletions to targeted insertions at high efficiencies.

After lunch and poster viewing, the first afternoon session was comprised of six talks selected from submitted abstracts. Gary Kucera of Duke University (Durham, NC, USA) described an approach to streamline the process of large targeting vector construction for complex gene modifications in ES cells. This was done using techniques that allow efficient CRISPR-mediated targeting with targeting vectors containing short homology arms or with the HITI (homology-independent targeted integration) method. Kazuto Yoshimi of Osaka University (Osaka, Japan) described their efficient CRISPR-mediated targeted knock-in of large constructs using lssDNA and the 2-hit-2-oligo (2H2O) strategy, the latter allowing targeted insertion of BAC constructs (as large as 200 kb) in rats. Denise Lanza of Baylor College of Medicine (Houston, TX, USA) presented the ongoing high-throughput conditional knock-out allele generation pipeline of IMPC using single-strand oligo DNA (ssODN) with CRISPR. Their quality control analysis indicated substantial frequency of random integration of ssDNA sequences, suggesting that attention must be paid when using ssODN in targeting. Rachel Delston of Canopy Biosciences (St. Louis, MO, USA) spoke about an innovative way to titrate expression levels of a given gene by introducing polyA tracks into its coding region by CRISPR, the method she referred to as TUNR. Katharina Boroviak of Welcome Trust Sanger Institute (Hinxton, UK) reported a follow up of their pipeline of CRISPR-assisted mutant allele generation in zygotes, including large deletions and inversions. Based on their experience, she cautioned the need to perform thorough primary analysis of targeted loci in order to gain full understanding of the allele variance in F0 founder animals and their derivatives. The session concluded with the presentation by Søren Warming of Genentech (South San Francisco, CA, USA) on in-depth analysis of CRISPR off-target effects in gene-edited rodents. Their next generation sequencing analysis indicated an alarming frequency of off-target incidences with up to 23% of the projects showing such events. He suggested that engineered high-fidelity Cas9 nucleases could be beneficial to avoid potential complications.

The last session of the day focused on new developments in genome editing and their applications. The first talk by Gaetan Burgio of Australian National University (Canberra, Australia) addressed recent examples of newly emerged reagents and techniques, such as NgAgo and the use of 2-guides 2-ssODN, both inspired much excitement in the community but later generated confusion and disappointment. He emphasized the importance of sharing results in the community, especially negative data, and even through social media networks, to avoid unnecessary commitment of community’s effort and resources that could result from questionable reagents and methodologies. Next Maria Jasin of Memorial Sloan Kettering Cancer Center (New York, USA) described the molecular mechanisms, particularly involving BRCA, in the understanding of how homologous recombination (HR) plays an integral part in the DNA repair pathway. Indeed, it is estimated that as much as 40% of DNA double strand breaks might be repaired by mechanisms involving HR in somatic cells. In our community’s interest, in depth mechanistic insights into HR repair in germline and zygotes may also aid in further improvements of targeted knock-in approaches using genome editing tools. Tony Nolan of Imperial College London (London, UK) described an application of CRISPR technology in mosquitos. His group has utilized synthetic homing endonucleases that enable properly designed alleles to be transmitted to offspring in a dominant manner. This interesting system, called gene drive, is based on a design that permits mutagenic Cas9/gRNA elements, when inserted into certain target loci, to become rapidly fixed to homozygosity and spread into the population. This can be applied to suppress certain insect populations, as in his studies involving Malaria parasite vectors. In more recent model populations the successful gene drive allele performed as designed in early generations, however, loss of gene drive was often found at later generations. This was due to the emergence of novel target site mutations resulting in resistance to the designed gene drive machinery. This finding poses future challenges towards the release of this system into wild mosquito populations. The final talk of the day was by Lauryl Nutter from the Center of Phenogenomics (Toronto, ON, Canada), summarizing the performance of their well-established CRISPR mutant generation pipeline. She described a variety of technical optimizations, including CRISPR applications in zygote injection and improved electroporation techniques. Their meticulous approaches to troubleshooting and quality control provided an excellent standard that can be widely referenced by the larger TT community. The evening concluded with the customary Gala Dinner where delegates enjoyed Western-themed country music, line dancing, and fine cuisine as well as a surprise celebration of Dr. Capecchi’s birthday.

The first morning session of the last Meeting day covered the topic of using animal models to understand human diseases. Zhongde Wang of Utah State University (Logan, UT, USA) emphasized the importance of non-mouse models; in his case, the golden Syrian hamster which exhibits a number of favorable attributes that more faithfully mimic various human diseases than do mouse models. He developed approaches for hamster embryo manipulation, applying CRISPR and Piggybac technologies for transgenesis. The next presentation by Monica Justice of the Hospital for Sick Children (Toronto, ON, Canada), by contrast, exemplified the power of mouse genetics to model human disease. The gene of her interest is *Mecp2*, whose human counterpart is implicated in Rett Syndrome. Her team conducted an elegant suppressor genetic screen in *Mecp2* mutant mice, and identified a genetic pathway of cholesterol metabolism that genetically interacts with Mecp2 to modulate Rett Syndrome-like symptoms in mice. Another study of human disease modeling in mice was presented by Indira Mysorekar of Washington University School of Medicine (St. Louis, Mo, USA) on Zika virus infections during pregnancy. Her team found that maternal inoculation of Zika virus during early pregnancy in mice appears to target both maternal and fetal placenta causing fetal death, a finding consistent with trans-placental infection. She demonstrated the validity of utilizing neutralizing antibodies to Zika virus to suppress placental and fetal virus infections in a mouse model, opening a possible venue for therapeutic use of vaccines in protection of fetuses from the disease. Large-scale drug discovery approaches using another well characterized genetic model system, zebrafish, were described by Randy Peterson of University of Utah (Salt Lake City, UT, USA). His talk clearly illustrated a number of powerful features of small chemical screening approaches in zebrafish, such as control of timing and dosage, and the direct assessment of efficacy and toxicity of given compounds. In combination with genetic tools, an impressive array of screening systems to identify biologically relevant pathways in development, neurophysiology and drug addictive behaviors were discussed.

The subsequent morning session highlighted techniques to facilitate phenotypic characterization of genetically engineered animal models. Nils Lindstrom of University of Southern California (Los Angeles, CA, USA) presented sophisticated live imaging techniques using organ culture system for phenotypic analyses of mouse and human kidney development. Dario Valenzano of Max Planck Institute for Biology of Aging (Cologne, Germany) introduced his approach using African turquoise killifish that have characteristic feature of diapause and are amenable to transgenic manipulation to study the mechanisms of aging. His recent studies showed a clear influence of the microbiota in the lifespan of this species. Charlotte D’Hulst of MouSensor, LLC (Brooklyn, NY, USA) developed an innovative transgenic mouse system, which she referred to as super sniffer mice, that enables predominant expression of a single odorant receptor transgene in the olfactory epithelium. Her approach can be applied to the development of mouse tools that aid in identification of certain odors associated with pre-symptomatic disease patients, such as those with Parkinson’s Disease, and perhaps may lead to discovery of novel disease associated biomarkers. The session was concluded by an exciting presentation of John Wiseman of AstraZeneca R&D (Mölndal, Sweden) on their pilot application of therapeutic CRISPR gene editing. He showed successful reversion of the liver damage phenotype in a humanized disease mouse model of human alpha-1-antitrypsin deficiency.

Continuing the new tradition initiated in TT2016 Meeting at Prague, this year’s Orbis Pictus Keynote Lecture was given by Martin Cohn of University of Florida, Gainsville (FL, USA) whose work focuses on the development and evolutionary origins of external genitalia, and mechanisms of sexual dimorphisms. He first summarized how conserved developmental genes regulate sexually dimorphic and evolutionary diverse morphogenetic features of external genitalia. He then described an integrated view on how different aspects of sexual dimorphisms in the human body, such as external genitalia, digit ratios, and certain brain nuclei that influence sexual behaviors, might be differentially regulated under a common androgen receptor signaling. Another TT Meeting tradition, ISTT Young Investigator Award presentation and Lecture, followed Cohn’s talk. The 5th ISTT Young Investigator Award, sponsored by inGenious Targeting Laboratory, went to Alexis Komor of Harvard University (Somerville, MA, USA) for her outstanding contributions to the development of new tools for genome editing. Her strategy was to develop Cas9 variants as DNA-modifying catalysts for precision base editing utilizing information from protein structural analysis. Her work was an impressively meticulous mechanistic approach to design useful molecular tools, and further improvement of such tools are yet to come from her new laboratory at UCSD.

As the final highlight of the TT2017 Meeting, Best Poster Awards selected by a committee of participating ISTT members were announced and presented. The three awardees this year were Alewo Idoko-Akoh of University of Edinburgh (Midlothian, Scotland, UK) for a precision CRISPR/Cas9 genome editing in chickens, Lisa Garrett of NIH/NHGRI (Bethesda, MD, USA) for the RNA-guided AsCpf1 nuclease for knock-in mouse generation, and Laramie Pence of University of Maryland (Silver Spring, MD, USA) for their study of long non-coding RNA in mouse placenta using CRISPR approaches. These outstanding presentations represented contributions to “technology refinement”, “new technology”, and “basic science breakthrough”, respectively. Congratulations to them all. The Meeting ended with a presentation for the next TT Meeting to be held in Kobe, a lively metropolitan city in west-central Japan, by Yas Furuta of RIKEN Center for Life Science Technologies (Kobe, Japan).

It should also be noted that the TT Meeting this year was preceded by two hands-on workshops, one on CRISPR technologies in mice (led by Channabasavaiah Gurumurthy (Guru) of University of Nebraska) and one for zebrafish embryo manipulation (led by Kristen Kwan of University of Utah), at the University of Utah. The venues for both of these workshops were filled with enthusiastic participants eager to learn and explore current and new technologies. All participants enjoyed their experience, and were pleased with this excellent learning opportunity.

Overall the meeting was another great success, well deserving of the applause given following the concluding remarks by Susan Tamowski. Delegates’ feedback was very positive on all aspects of the Meeting, from excellent talks, active participation by attendees in scientific and social programs, to the superb management by the University of Utah Conference & Event Management department, as well as the effort of the Organizing Committee behind the scene. The weather on the final afternoon was beautiful, accentuating the majestic scenery of the valley to further stimulate the inspiration of the delegates celebrating the completion of the Meeting. This trend of creating such extraordinary occasions for meeting delegates should be continued for future TT Meetings with the next in Kobe coinciding with the beautiful cherry blossom season. We invite and encourage all scientists and research staff in the field of transgenic research and sponsors from all around the world to attend and enjoy the TT2019 Meeting in Japan.

